# Inpatient multidisciplinary care can prevent deterioration of renal function in patients with chronic kidney disease: a nationwide cohort study

**DOI:** 10.3389/fendo.2023.1180477

**Published:** 2023-06-20

**Authors:** Masanori Abe, Tsuguru Hatta, Yoshihiko Imamura, Tsutomu Sakurada, Shinya Kaname

**Affiliations:** ^1^ The Committee of the Evaluation and Dissemination for Certified Kidney Disease Educator, Japan Kidney Association, Tokyo, Japan; ^2^ Division of Nephrology, Hypertension and Endocrinology, Department of Internal Medicine, Nihon University School of Medicine, Tokyo, Japan; ^3^ Department of Medicine, Hatta Medical Clinic, Kyoto, Japan; ^4^ Department of Nephrology, Nissan Tamagawa Hospital, Tokyo, Japan; ^5^ Department of Nephrology and Hypertension, St. Marianna University School of Medicine, Kawasaki, Kanagawa, Japan; ^6^ Department of Nephrology and Rheumatology, Kyorin University School of Medicine, Tokyo, Japan

**Keywords:** certified kidney disease educator, chronic kidney disease, estimated glomerular filtration rate, inpatient educational program, multidisciplinary care, outpatient guidance, renal replacement therapy

## Abstract

**Background:**

Multidisciplinary care is necessary to prevent worsening renal function and all-cause mortality in patients with chronic kidney disease (CKD) but has mostly been investigated in the outpatient setting. In this study, we evaluated the outcome of multidisciplinary care for CKD according to whether it was provided in an outpatient or inpatient setting.

**Methods:**

This nationwide, multicenter, retrospective, observational study included 2954 Japanese patients with CKD stage 3–5 who received multidisciplinary care in 2015–2019. Patients were divided into two groups: an inpatient group and an outpatient group, according to the delivery of multidisciplinary care. The primary composite endpoint was the initiation of renal replacement therapy (RRT) and all-cause mortality, and the secondary endpoints were the annual decline in the estimated glomerular filtration rate (ΔeGFR) and the changes in proteinuria between the two groups.

**Results:**

Multidisciplinary care was provided on an inpatient basis in 59.7% and on an outpatient basis in 40.3%. The mean number of health care professionals involved in multidisciplinary care was 4.5 in the inpatient group and 2.6 in the outpatient group (P < 0.0001). After adjustment for confounders, the hazard ratio of the primary composite endpoint was significantly lower in the inpatient group than in the outpatient group (0.71, 95% confidence interval 0.60-0.85, P = 0.0001). In both groups, the mean annual ΔeGFR was significantly improved, and proteinuria significantly decreased 24 months after the initiation of multidisciplinary care.

**Conclusion:**

Multidisciplinary care may significantly slow deterioration of eGFR and reduce proteinuria in patients with CKD and be more effective in terms of reducing initiation of RRT and all-cause mortality when provided on an inpatient basis.

## 1 Introduction

Increasing numbers of patients have chronic kidney disease (CKD) worldwide (1). In Japan, nearly 15 million adults were estimated to have CKD in 2015 (2), and increasing numbers of patients with end-stage kidney disease are starting renal replacement therapy (RRT) each year, with more than 340,000 patients now receiving dialysis (3). The prevalence of dialysis in Japan is 2682 per million population, second only to Taiwan (4). A comprehensive approach to management is needed because CKD increases the risk of not only ESKD but also cardiovascular mortality. Thus it is necessary to control blood pressure, glycemic status, anemia, bone mineral status, and low-density lipoprotein cholesterol alongside lifestyle modification, dietary guidance, and measures to ensure adherence with medication (5, 6). It has been reported that comprehensive multidisciplinary care can reduce all-cause mortality, the likelihood of temporary catheterization for patients on dialysis, and the hospitalization rate as well as slow decline in the estimated glomerular filtration rate (eGFR) (7–10). In these studies, comprehensive multidisciplinary care was provided by teams that included nephrologists, specialist nurses, dieticians, pharmacists, and social workers.

The Certified Kidney Disease Educator (CKDE) system was established in Japan by the Japan Kidney Association in 2017 with the aims of preventing progression of CKD and improving and maintaining quality of life for patients with CKD. Nurses, registered dieticians, and pharmacists who meet certain requirements are eligible to qualify as a CKDE. All CKDEs have acquired the basic skills for managing patients with CKD, including providing guidance on lifestyle modification, dietary counseling, and medical therapy according to disease stage. Generally, multidisciplinary care for patients with CKD and diabetes is performed on an outpatient basis, as reflected in the Steno-2 and MASTERPLAN studies (11–14). However, in Japan, widespread multidisciplinary care for patients with CKD is provided not only on an outpatient basis but also on an inpatient basis because of lack of time during outpatient appointments to cover lifestyle modification, dietary restriction, and medication adherence in sufficient depth. Currently, however, there is limited information on whether these multidisciplinary interventions in the inpatient setting improve the prognosis of CKD.

We conducted this nationwide study to assess the outcome of multidisciplinary intervention in patients with CKD according to whether it was provided in an outpatient or inpatient setting.

## 2 Methods

### 2.1 Study design and participants

This nationwide multicenter retrospective cohort study was performed by members of the Japan Kidney Association Committee for Evaluation and Dissemination of CKDE. To reflect practice patterns across most of Japan, around 3000 Japanese patients were participated at any of 24 selected health care institutions in Japan that play a central role in the treatment of patients with CKD. All-cause mortality and the start of RRT were tracked until the end of 2020 for patients with CKD who had data on kidney function available for the 12 months before to and 24 months after receiving multidisciplinary therapy between January 2015 and December 2019. The following exclusion requirement were used: age < 20 years; CKD stage 1 and 2 (i.e., eGFR ≥ 60 mL/min/1.73 m^2^); patients who were hospitalized for another reason other than CKD; short-term follow-up of 6 months or less; received multidisciplinary care in the past; active malignant disease; transplant recipient; history of long-term dialysis; and data missing for age, sex, kidney function, or results. In Japan, multidisciplinary care for patients with CKD was conducted in outpatient or inpatient settings based on the hospital functions, nephrologists’ judgment, and the patient’s wishes. As a result, the enrolled patients were classified into an outpatient and an inpatient group based on the approach and place of intervention by the multidisciplinary care team at the start of the intervention (baseline). They were further divided into subgroups based on whether they had diabetes. A group of inpatient patients were admitted to the hospital and received multidisciplinary care in accordance with each facility’s inpatient educational program.

The main efficacy composite endpoint was the initiation of RRT and all-cause mortality at the end of 2020. The secondary efficacy endpoint was the annual decline in eGFR (ΔeGFR) and the annual change in urinary protein level between 12 months before and 6, 12, and 24 months after the initiation of multidisciplinary intervention.

The study was approved by the ethics committee of Nihon University Itabashi Hospital and conducted in accordance with the Declaration of Helsinki, Japanese privacy protection laws, and the Ethical Guidelines for Medical and Health Research Involving Human Subjects published by the Ministry of Education, Culture, Sports, Science and Technology and the Ministry of Health, Labour and Welfare in 2015. The need for informed consent was waived in view of the use of de-identified data. The study is registered in the University Hospital Medical Information Network (UMIN000049995).

### 2.2 Multidisciplinary care

The definition of multidisciplinary care adopted was (1) a multidisciplinary care team composed of nephrologists and other professionals (i.e., specialist nurses, registered dieticians, pharmacists, physical therapists, social workers, clinical engineers, and clinical laboratory technicians) and (2) an operational model of multidisciplinary care comprising patient education, medical management, and lifestyle modification according to CKD stage. The quality of the educational content provided was maintained based on the text created by the Japanese Society of Nephrology, the Japanese Society for Dialysis Therapy, the Japan Society for Transplant, and the Japanese Society for Clinical Renal Transplantation or the CKD Teaching Guidebook for Certified Kidney Disease Educators published by the Japan Kidney Association (15, 16).

### 2.3 Data collection

Patient demographics and data on clinical characteristics were collected, including age, sex, history of cardiovascular disease (CVD), primary etiology of CKD, body mass index (BMI), hemoglobin, serum albumin, urea nitrogen, creatinine (Cr), eGFR, and urinary protein. Information on glycated hemoglobin (HbA1c) was also collected for patients with diabetes at baseline. CVD was defined as coronary artery disease, ischemic stroke, hemorrhagic stroke, or limb amputation. eGFR was calculated according to the following formula for Japanese patients: eGFR (mL/min/1.73 m^2^) = 194 × serum Cr^−1.094^ × age^−0.287^ (× 0.739 for women) (17). Urinary protein was calculated as the urinary protein to Cr ratio (UPCR). The data on method and place of intervention (outpatient or inpatient), the number or duration of interventions (number of visits for outpatient intervention or the number of hospitalization days for inpatients), and the type and number of health care professionals involved in the multidisciplinary care team were also collected. Data were collected for the primary composite endpoint, which included the date attained or the end of 2020, whichever came first (initiation of RRT and all-cause mortality). Also noted was the RRT’s kind (kidney transplantation, peritoneal dialysis, or hemodialysis).

### 2.4 Statistical analysis

Data are reported as the number and proportion, mean ± standard deviation, or median [interquartile range] as appropriate. Intragroup comparisons were made using two-tailed paired t-tests. Categorical variables were examined using the chi-squared test and continuous variables using the t-test. The composite outcome was estimated using the Kaplan–Meier method and compared between groups using the log-rank test. A univariate analysis was performed according to the method and place of intervention (i.e., outpatient-based or inpatient-based). Multivariate survival analyses were performed using Cox proportional hazards models with adjustment for confounding factors to examine the method and place of intervention and the composite outcome during the 6 years of follow-up. Model 1 was used to calculate hazard ratios (HRs) adjusted for basic factors, including age, sex, history of CVD, eGFR, and UPCR at baseline, and model 2 was adjusted for BMI, hemoglobin, and serum albumin level in addition to the factors included in model 1. A subgroup analysis was performed according to the diabetes status and the CKD stage (G3a, G3b, G4, or G5) at baseline. A further subdivision analysis in the inpatient group based on the presence or absence of physical therapists was performed. In patients with diabetes, model 1 was used to calculate the HRs with adjustment for basic factors (e.g., age, sex, history of CVD, HbA1c, eGFR, and UPCR at baseline), and model 2 was adjusted for BMI, hemoglobin, and serum albumin level in addition to the factors included in model 1. In patients without diabetes, model 1 was used to calculate the HRs adjusted for basic factors, including age, sex, history of CVD, eGFR, and UPCR at baseline and model 2 was adjusted for BMI, hemoglobin, and serum albumin level in addition to the factors included in model 1. The results from the models are reported as HRs with 95% confidence intervals (CIs) and P-values. For the regression analyses, imputation of missing data was performed by conventional methods as appropriate. All analyses were performed using JMP^®^ version 13.0 (SAS Institute Inc., Cary, NC, USA). Statistical significance was set at P-values less than 0.05.

## 3 Results

### 3.1 Patient characteristics at time of initiation of multidisciplinary care

Overall, of the 3296 patients enrolled, 342 were removed (CKD stage 1 or 2, n = 118; age younger than 20 years, n = 3; follow-up for 6 months or less, n = 124; lack of data for baseline kidney function, n = 13), which left 2954 patients for inclusion in the analysis. Patient characteristics at the time of initiation of multidisciplinary care are shown in **Table 1**. The mean age was 70.5 ± 11.6 years, and 74.1% of the patients were male. The mean eGFR was 26.3 ± 12.5 mL/min/1.73 m^2^ and the median UPCR was 1.09 g/gCr [0.23, 2.98]. The most common etiology of CKD was diabetic kidney disease (42.7%), followed by nephrosclerosis (30.8%) and chronic glomerulonephritis (12.6%). The most common CKD stage was G4 (42.4%), followed by G3b (26.1%) and G5 (21.8%).

**Table 1:** Baseline characteristics of all study participants.

Variable	
Patients, n (% male)	2954 (74.1)
Age, years	70.5 ± 11.6
Body mass index	24.2 ± 4.3
Serum creatinine, mg/dL	2.02 [1.46, 3.02]
eGFR, mL/min/1.73 m^2^	26.3 ± 12.5
Serum urea nitrogen, mg/dL	31 [23–43]
Hemoglobin, g/dL	11.7 ± 1.9
Serum albumin, g/dL	3.8 ± 0.5
Urinary protein, g/gCr	1.09 [0.23, 2.98]
Comorbid CVD, n (%)	846 (28.6)
HbA1c (in patients with diabetes), %	6.4 ± 1.0
Primary cause of CKD, n (%)	
Diabetic kidney disease	1263 (42.7)
Nephrosclerosis	909 (30.8)
Chronic glomerulonephritis	374 (12.6)
Polycystic kidney disease	87 (3.0)
Other	321 (10.9)
CKD stage, n (%)	
G3 (G3a + G3b)	1059 (35.9)
G3a	288 (9.8)
G3b	771 (26.1)
G4	1251 (42.4)
G5	644 (21.8)
Number of professionals on MDC team, n (%)	
Total number of professionals on MDC team, n	3.8 ± 1.2
2	656 (22.2)
3	398 (13.5)
4	902 (30.5)
5	976 (33.0)
6	22 (0.8)
Members of MDC team, n (%)	
Nurses	2545 (86.2)
Registered dieticians	2703 (91.5)
Pharmacists	1885 (63.8)
Physical therapists	772 (26.1)
Clinical laboratory technicians	171 (5.8)
Social workers	68 (2.3)
Others	24 (0.8)

Data are shown as the number (percentage), mean ± standard deviation, or median [interquartile range] as appropriate. CKD, chronic kidney disease; CVD, cardiovascular disease; eGFR, estimated glomerular filtration rate; HbA1c, glycated hemoglobin; MDC, multidisciplinary care.

### 3.2 Type and number of professionals in the multidisciplinary care team

Details of the interventions implemented by the multidisciplinary care team are shown in **Table 1**. The mean number of multidisciplinary care team members, including nephrologists, was 3.8 ± 1.2. It was most common for the multidisciplinary care team to include five professionals (33.0%), followed by four (30.5%) and the two (22.2%). Registered dieticians were the most common members of the multidisciplinary care team (91.5%), followed by specialist nurses (86.2%), pharmacists (63.8%), and physical therapists (26.1%).

### 3.3 Outcomes

The median observation period was 36 months [22, 52], during which 128 patients (4.3%) died, 648 (21.9%) initiated RRT, and 66 (2.2%) were lost to follow-up; 2112 (71.6%) of all patients were alive without RRT at the end of the study period. RRT consisted of hemodialysis in 559 patients (86.2%), peritoneal dialysis in 66 (10.2%), and kidney transplantation in 23 (3.6%).

#### 3.3.1 Comparison between outpatient and inpatient groups

The baseline characteristics of the patients in the inpatient and outpatient groups are shown in **Table 2**. Intervention was provided in an inpatient setting for more than half of the patients (59.7%) and on an outpatient basis for the remainder (40.3%). The baseline kidney function, including eGFR, serum Cr and UPCR, was comparable between the two groups, but patients in the inpatient group were more likely to be female and older and to have a higher BMI and comorbid CVD. However, rates of diabetic kidney disease and CKD stage G5 were lower in the inpatient group than in the outpatient group. The mean number of multidisciplinary care team members was significantly higher in the inpatient group (4.5 ± 0.6 *vs*. 2.6 ± 0.7, P < 0.0001).

**Table 2:** Baseline characteristics in the outpatient and inpatient groups.

Variable	Outpatient group	Inpatient group	P-value
Patients, n (% male)	1190 (79.3)	1764 (70.6)	< 0.0001
Age, years	69.6	71.2	0.0004
Body mass index	23.6 ± 3.9	24.5 ± 4.4	< 0.0001
Serum creatinine, mg/dL	2.08 [1.45, 3.16]	1.99 [1.47, 2.93]	0.165
eGFR, mL/min/1.73 m^2^	26.1 ± 12.9	26.4 ± 12.3	0.786
Serum urea nitrogen, mg/dL	32 [23, 45]	31 [23, 42]	0.239
Hemoglobin, g/dL	11.8 ± 1.9	11.7 ± 1.9	0.123
Serum albumin, g/dL	3.8 ± 0.5	3.7 ± 0.5	< 0.0001
Urinary protein, g/gCr	1.20 [0.27, 3.25]	1.01 [0.22, 2.87]	0.218
Comorbid CVD, n (%)	334 (28.1)	512 (29.0)	< 0.0001
HbA1c (in patients with diabetes), %	6.4 ± 0.9	6.4 ± 1.1	0.188
Primary cause of CKD, n (%)			< 0.0001
Diabetic kidney disease	579 (48.6)	684 (38.8)	
Nephrosclerosis	259 (21.8)	650 (36.8)	
Chronic glomerulonephritis	126 (10.6)	248 (14.0)	
Polycystic kidney disease	45 (3.8)	42 (2.4)	
Others	321 (15.2)	140 (8.0)	
CKD stage, n (%)			0.005
G3 (G3a + G3b)	431 (36.2)	624 (35.6)	
G3a	129 (10.8)	159 (9.0)	
G3b	302 (25.4)	469 (26.6)	
G4	469 (39.4)	782 (44.3)	
G5	290 (24.4)	354 (20.1)	
Number of interventions, n or days	4 [1, 10]	7 [5, 12]	—
Total number of professionals on MDC team, n	2.6 ± 0.7	4.5 ± 0.6	< 0.0001
Number of professionals on MDC team, n (%)			< 0.0001
2	641 (53.9)	17 (1.0)	
3	363 (30.5)	33 (1.9)	
4	178 (15.0)	724 (41.0)	
5	6 (0.5)	970 (55.0)	
6	2 (0.1)	20 (1.1)	
Members of MDC team, n (%)			
Nurses	790 (66.4)	1755 (99.5)	< 0.0001
Registered dieticians	948 (79.6)	1755 (99.5)	< 0.0001
Pharmacists	172 (14.5)	1713 (97.1)	< 0.0001
Physical therapists	0 (0)	772 (43.8)	< 0.0001
Clinical laboratory technicians	0 (0)	171 (9.7)	< 0.0001
Social workers	5 (0.4)	63 (3.6)	< 0.0001
Others	21 (1.8)	3 (0.2)	< 0.0001

Data are shown as the number (percentage), mean ± standard deviation, or median [interquartile range] as appropriate. CKD, chronic kidney disease; CVD, cardiovascular disease; eGFR, estimated glomerular filtration rate; HbA1c, glycated hemoglobin; MDC, multidisciplinary care.

**Figure 1:**
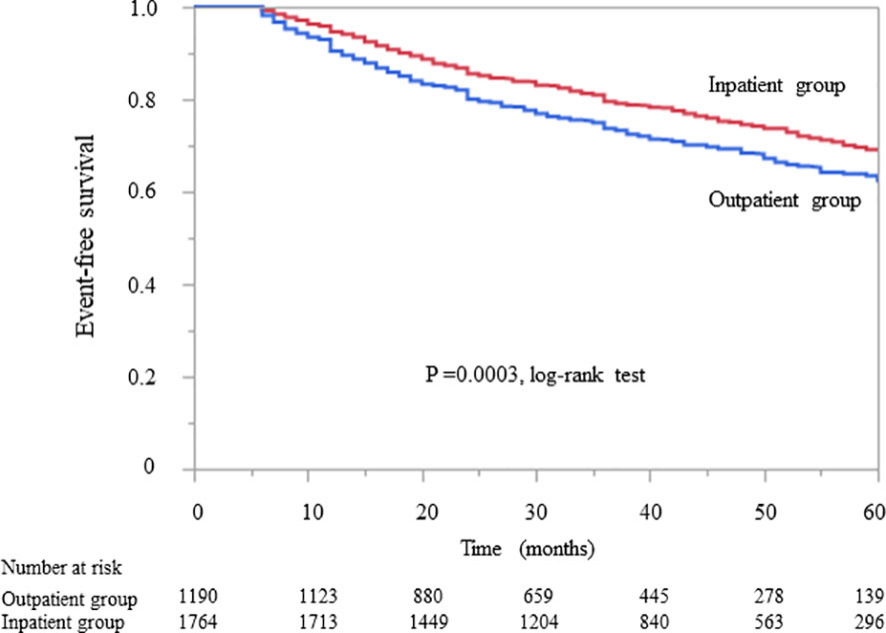
Kaplan–Meier curves showing the incidence of initiation of renal replacement therapy and all-cause mortality in Japanese patients with chronic kidney disease according to whether they received outpatient or inpatient multidisciplinary care.

Kaplan–Meier analysis for the composite endpoint (initiation of RRT and all-cause mortality) revealed a significant difference between the outpatient and inpatient groups (P = 0.0003, log-rank test; **Figure 1**). Compared with the outpatient (reference) group, the inpatient group had a significantly lower unadjusted HR for the composite endpoint (0.78, 95% CI 0.68–0.91, P = 0.0004). After adjustment for basic factors, including age, sex, history of CVD, eGFR, and UPCR at baseline, the HR in the inpatient group was 0.73 (95% CI 0.63–0.88, P = 0.0001). After further adjustment for basic factors and BMI, hemoglobin, and serum albumin at baseline, the HR was significantly lower in the inpatient group (0.71, 95% CI 0.60–0.85, P = 0.0001) (**Table 3**).

**Table 3:** Comparison of initiation of renal replacement therapy and all-cause mortality between the outpatient and inpatient groups in Cox proportional hazards models adjusted for confounding factors in Japanese patients with chronic kidney disease.

Group	Unadjusted			Model 1			Model 2		
	HR	95% CI	P-value	HR	95% CI	P-value	HR	95% CI	P-value
Outpatient	1.00	Reference	—	1.00	Reference	—	1.00	Reference	—
Inpatient	0.78	0.68–0.91	0.0004	0.73	0.63–0.88	0.0001	0.71	0.60–0.85	0.0001

Model 1 was adjusted for basic factors, including age, sex, history of cardiovascular disease, estimated glomerular filtration rate, and urinary protein level at baseline. Model 2 was adjusted for body mass index, hemoglobin, and serum albumin level at baseline in addition to the factors included in model 1. CI, confidence interval; HR, hazard ratio.

### 3.4 Subgroup analysis according to diabetes status

**Figure 2:**
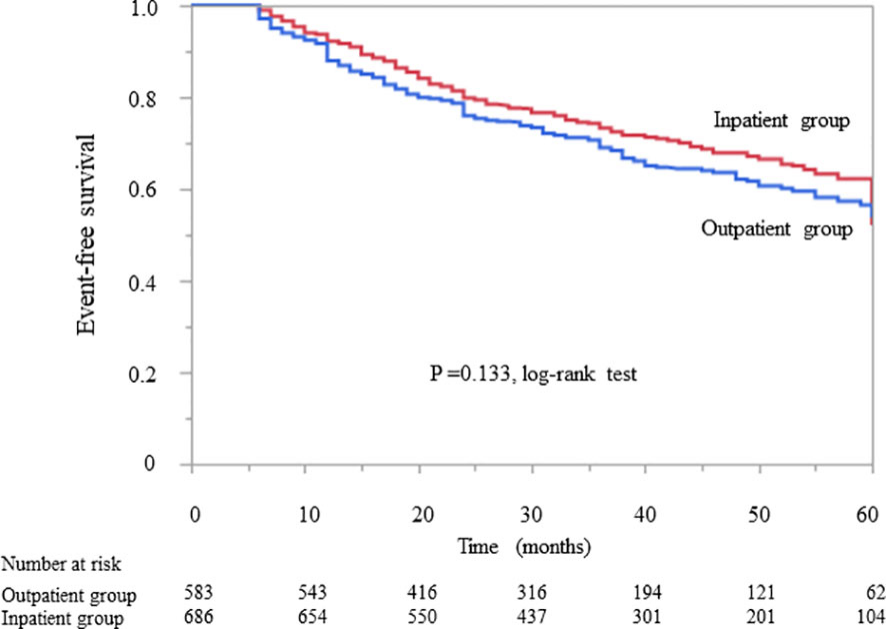
Kaplan–Meier curves showing the incidence of initiation of renal replacement therapy and all-cause mortality in Japanese diabetes patients with chronic kidney disease according to whether they received outpatient or inpatient multidisciplinary care.

Kaplan–Meier analysis revealed that there was no significant difference in the composite endpoint between patients with diabetes in the outpatient group and those in the inpatient group (P = 0.133, log-rank test; **Figure 2**). Cox proportional analysis revealed no significant difference in the unadjusted HR for the composite endpoint between the inpatient and outpatient groups (**Table 4**). However, after adjustment for basic factors, including age, sex, history of CVD, HbA1c, eGFR, and UPCR at baseline, the HR in the inpatient group was 0.75 (95% CI 0.61–0.93, P = 0.010). After further adjustment for basic factors and BMI, hemoglobin, and serum albumin level at baseline, the inpatient group had a significantly lower HR (0.74, 95% CI 0.59–0.95, P = 0.018) (**Table 4**).

**Table 4:** Comparison of initiation of renal replacement therapy and all-cause mortality between the outpatient and inpatient groups in Cox proportional hazards models adjusted for confounding factors in Japanese patients with chronic kidney disease and diabetes.

Group	Unadjusted			Model 1			Model 2		
	HR	95% CI	P-value	HR	95% CI	P-value	HR	95% CI	P-value
Outpatient	1.00	Reference	—	1.00	Reference	—	1.00	Reference	—
Inpatient	0.86	0.71–1.05	0.138	0.75	0.61–0.93	0.01	0.74	0.59–0.95	0.018

Model 1 was adjusted for basic factors, including age, sex, history of cardiovascular disease, glycated hemoglobin, estimated glomerular filtration rate, and urinary protein level at baseline. Model 2 was adjusted for body mass index, hemoglobin, and serum albumin at baseline in addition to the factors included in model 1. CI, confidence interval; HR, hazard ratio.

**Figure 3:**
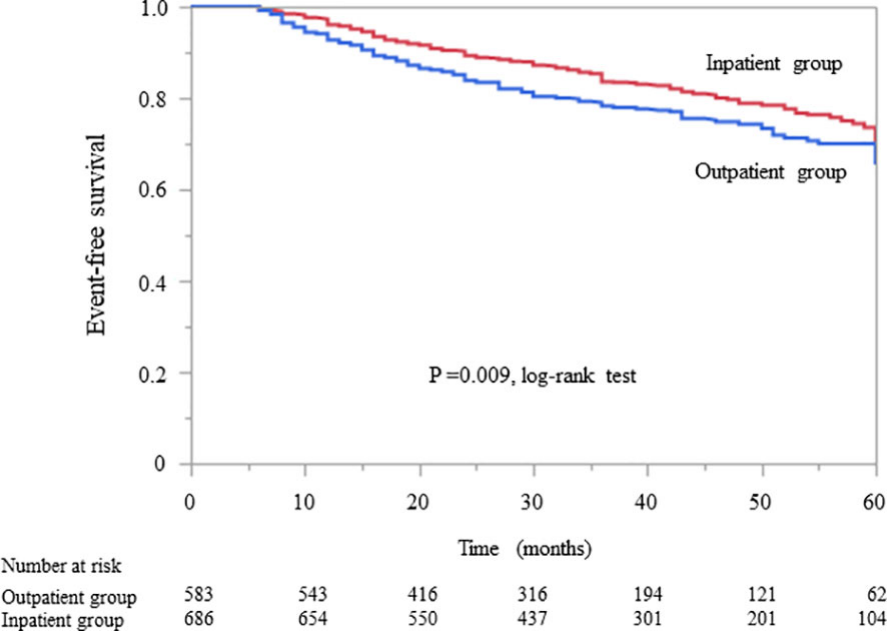
Kaplan–Meier curves for the incidence of initiation of renal replacement therapy and all-cause mortality in Japanese non-diabetes patients with chronic kidney disease in the outpatient and inpatient groups.

In patients without diabetes, Kaplan–Meier analysis revealed a significant difference in the composite endpoint between the outpatient and inpatient groups (P = 0.009, log-rank test; **Figure 3**). Compared with the outpatient group, the inpatient group had a significantly lower unadjusted HR for the composite endpoint (0.75, 95% CI 0.61–0.93, P = 0.009). After adjustment for basic factors, including age, sex, history of CVD, eGFR, and UPCR at baseline, the HR in the inpatient group was 0.75 (95% CI 0.59–0.94, P = 0.015). After further adjustment for basic factors and BMI, hemoglobin, and serum albumin level at baseline, the inpatient group had a significantly lower HR (0.76, 95% CI 0.59–0.98, P = 0.034) (**Table 5**).

**Table 5:** Comparison of all-cause mortality between the outpatient and inpatient groups according to Cox proportional hazards models adjusted for confounding factors in Japanese patients with chronic kidney disease but no diabetes.

Group	Unadjusted			Model 1			Model 2		
	HR	95% CI	P value	HR	95% CI	P value	HR	95% CI	P value
Outpatient	1.00	Reference	—	1.00	Reference	—	1.00	Reference	—
Inpatient	0.75	0.61–0.93	0.009	0.75	0.59–0.94	0.015	0.76	0.59–0.98	0.034

Model 1 was adjusted for basic factors, including age, sex, history of cardiovascular disease, estimated glomerular filtration rate, and urinary protein level at baseline. Model 2 was adjusted for body mass index, hemoglobin, and serum albumin at baseline in addition to the factors included in model 1. CI, confidence interval; HR, hazard ratio.

### 3.5 Subgroup analysis according to the CKD stage at baseline

**Figure 4:**
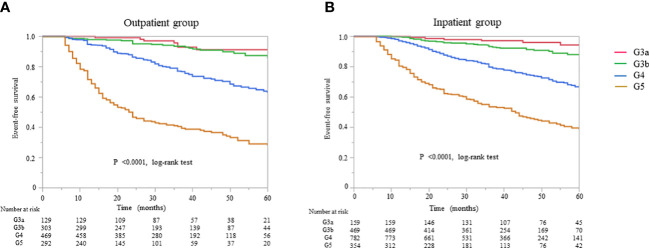
Kaplan–Meier curves for the incidence of initiation of renal replacement therapy and all-cause mortality in Japanese patients with chronic kidney disease according to the baseline stages in the **(A)** outpatient and **(B)** inpatient groups.

All-cause mortality and RRT initiation were dependent on the disease stage. The Kaplan–Meier analysis revealed that the composite endpoint varied significantly depending on the CKD stage at baseline in both groups (P < 0.0001, log-rank test; **Figure 4**). After the adjustment of basic factors, including age, sex, comorbid CVD, and the presence or absence of diabetes, the HRs in the G3b, G4, and G5 groups were compared with the G3a (reference) group and were significantly higher in both. However, after the adjustment of basic factors and laboratory data, including BMI, hemoglobin, serum albumin, and UPCR level, the G4 and G5 groups had significantly higher HRs (**Tables 6**, **7**).

**Table 6:** All-cause mortality and initiation of renal replacement therapy according to the CKD stage at baseline in Cox proportional hazards models adjusted for confounding factors in the outpatient group.

Group	Unadjusted			Model 1			Model 2		
	HR	95% CI	P-value	HR	95% CI	P-value	HR	95% CI	P-value
G3a	1.00	Reference	—	1.00	Reference	—	1.00	Reference	—
G3b	2.63	1.21–6.92	0.013	1.76	0.80–4.42	0.164	1.41	0.57–3.99	0.468
G4	7.87	3.82–20.0	<0.0001	5.65	2.83–13.4	<0.0001	3.65	1.67–9.59	0.001
G5	22.8	11.1–58.9	<0.0001	21.0	10.63–49.7	<0.0001	12.8	5.91–33.8	<0.0001

Model 1 was adjusted for basic factors, including age, sex, history of cardiovascular diseases, presence or absence of diabetes, and urinary protein levels at baseline. Model 2 was adjusted the same as Model 1 but with additional adjustments for body mass index, hemoglobin, and serum albumin levels at baseline. CI, confidence interval; CKD, chronic kidney disease; HR, hazard ratio.

**Table 7:** All-cause mortality and initiation of renal replacement therapy according to the CKD stage at baseline in Cox proportional hazards models adjusted for confounding factors in the inpatient group.

Group	Unadjusted			Model 1			Model 2		
	HR	95% CI	P-value	HR	95% CI	P-value	HR	95% CI	P-value
G3a	1.00	Reference	—	1.00	Reference	—	1.00	Reference	—
G3b	2.63	1.21–6.92	0.013	2.94	1.34–7.73	0.005	2.18	0.98–5.80	0.056
G4	7.87	3.82–20.0	<0.0001	9.08	4.38–23.1	<0.0001	5.58	2.64–14.3	< 0.0001
G5	22.8	11.1–58.9	<0.0001	27.9	13.5–71.5	<0.0001	15.2	7.10–39.8	< 0.0001

Model 1 was adjusted for basic factors, including age, sex, history of cardiovascular diseases, presence or absence of diabetes, and urinary protein levels at baseline. Model 2 was adjusted the same as Model 1 but with additional adjustments for body mass index, hemoglobin, and serum albumin levels at baseline. CI, confidence interval; CKD, chronic kidney disease; HR, hazard ratio.

### 3.6 Subgroup analysis based on the presence or absence of physical therapists in the inpatient group

**Figure 5:**
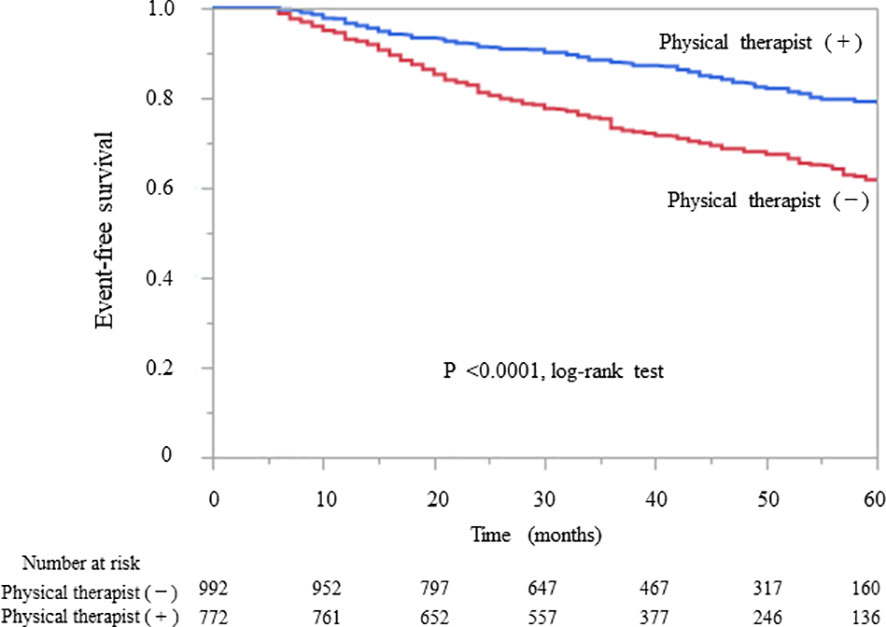
Kaplan–Meier curves for the initiation of renal replacement therapy and all-cause mortality in Japanese patients with chronic kidney disease based on the presence or absence of physical therapists in the inpatient subgroups.

The patients in the inpatient group were subdivided into two groups with and without a physical therapist in the multidisciplinary care team. The baseline characteristics of the two groups are shown in **Supplementary Table 1**. The group with physical therapists had higher eGFR and lower proteinuria at baseline, with a higher rate of comorbid CVD and diabetic kidney disease. The Kaplan–Meier analysis revealed a significant difference in the composite endpoint between the two groups (P < 0.0001, log-rank test; **Figure 5**). Compared with the group without physical therapists, the group with physical therapists had a significantly lower unadjusted HR for the composite endpoint (0.52, 95% CI 0.42–0.63, P < 0.0001). After the adjustment of basic factors, including age, sex, history of CVD, eGFR, and UPCR at baseline, the HR in the group with physical therapists was 0.51 (95% CI 0.41–0.64, P < 0.0001). After further adjustment of basic factors and BMI, hemoglobin, and serum albumin level at baseline, the group with physical therapists had a significantly lower HR (0.55, 95% CI 0.42–0.71, P < 0.0001) (**Supplementary Table 2**).

### 3.7 ΔeGFR and change in UPCR before and after multidisciplinary care in all patients

**Figure 6:**
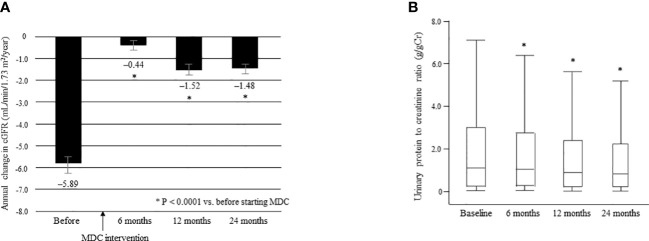
Annual change in eGFR in the 12 months before and 24 months after starting multidisciplinary care in all patients **(A)**. Data are shown as the mean. Bars indicate the 95% confidence interval. *P < 0.0001 *vs*. before start of MDC. Changes in the urinary protein level between time of initiation of MDC and 24 months later **(B)**. Data are shown as the median and interquartile range. *P < 0.0001 *vs*. baseline. ΔeGFR, change in eGFR; eGFR, estimated glomerular filtration rate; MDC, multidisciplinary care.

The mean ΔeGFR was significantly improved from –5.89 ± 7.17 before multidisciplinary intervention to –0.44 ± 5.21 at 6 months, –1.52 ± 6.09 at 12 months, and –1.48 ± 3.78 at 24 months after intervention (all P < 0.0001; **Figure 6A**). The median UPCR was significantly decreased from 1.09 g/gCr [0.23, 2.98] at baseline to 1.00 g/gCr [0.24, 2.71] at 6 months, 0.89 g/gCr [0.21, 2.38] at 12 months, and 0.82 g/gCr [0.20, 2.22] at 24 months (all P < 0.0001; **Figure 6B**).

#### 3.7.1 ΔeGFR and change in UPCR before and after multidisciplinary care in the two groups

**Figure 7:**
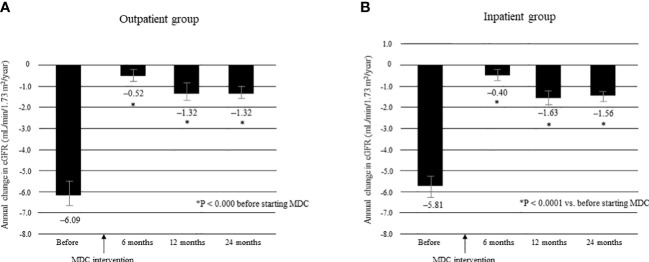
Annual change in eGFR in the 12 months before and 24 months after starting MDC in the outpatient group **(A)** and in the inpatient group **(B)**. *P < 0.0001 *vs*. before start of MDC. Data are shown as the mean. Bars indicate the 95% confidence interval. ΔeGFR, change in eGFR; eGFR, estimated glomerular filtration rate; MDC, multidisciplinary care.

The mean ΔeGFR before and after multidisciplinary intervention in each group is shown in **Figure 7**. There was no significant between-group difference in mean ΔeGFR before intervention (**Supplementary Table 3**). The mean ΔeGFR was -6.09 ± 7.65 before intervention and -0.52 ± 5.23 at 6 months, -1.32 ± 6.01 at 12 months, and -1.32 ± 3.64 at 24 months after intervention in the outpatient group (all P < 0.0001; **Figure 7A**); the respective values in the inpatient group were -5.81 ± 7.43, -0.40 ± 5.20, -1.63 ± 6.15, and -1.56 ± 3.84 (all P < 0.0001; **Figure 7B**). There was no significant between-group difference in mean ΔeGFR at any time point after intervention (**Supplementary Table 3**).

**Figure 8:**
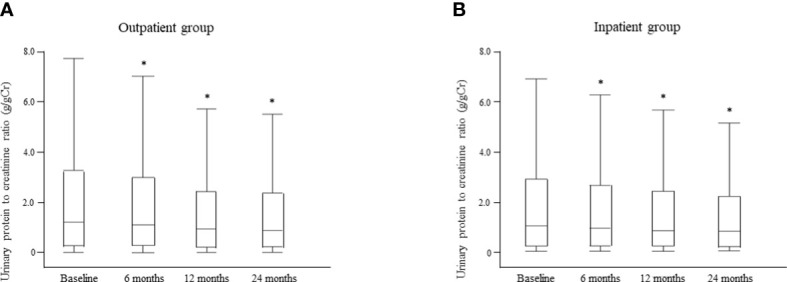
Changes in the urinary protein level between the time of starting multidisciplinary care and 24 months later in the outpatient **(A)** and inpatient **(B)** groups. Data are shown as the median and interquartile range. *P < 0.0001 *vs*. baseline.

Changes in the median UPCR after intervention by the multidisciplinary care team are shown for each group in **Figure 8**. There was no significant between-group difference in UPCR at baseline. However, in the outpatient group, the median UPCR decreased significantly from 1.20 g/gCr [0.27, 3.25] at baseline to 1.10 g/gCr [0.29, 2.98] at 6 months, 0.94 g/gCr [0.22, 2.42] at 12 months, and 0.88 g/gCr [0.24, 2.36] at 24 months (all P <0.0001; **Figure 8A**); the respective values in the inpatient group were 1.01 g/gCr [0.22, 2.87], 0.92 g/gCr [0.21, 2.61], 0.82 g/gCr [0.21, 2.37], and 0.79 g/gCr [0.17, 2.28] (all P < 0.0001; **Figure 8B**). Furthermore, there was no significant between-group difference in the median UPCR at any time point after intervention (**Supplementary Table 4**).

## 4 Discussion

This nationwide cohort study included 2954 individuals from 24 facilities in Japan. We found that patients with CKD currently receive multidisciplinary care more often in hospitals (59.7%) than in an outpatient setting (40.3%) in Japan. The major strengths of this study are its large sample population recruited from multiple centers, the relatively long observation period, and inclusion of a comparatively high number of elderly patients. Although the mean age of patients in the previous studies was younger than 70 years, our mean age was 70.5 years, reflecting our aging CKD population in Japan (5, 7–10). This study is the first to suggest that multidisciplinary care may be able to prevent worsening kidney function in Japanese patients with CKD regardless of whether it is provided on an outpatient or inpatient basis. The rate of RRT initiation and all-cause mortality over the longer observation period of 6 years were the key composite endpoints, and although there was no significant difference between the two groups’ baseline eGFR levels, there was a significant between-group difference in both variables. Therefore, our results suggest that multidisciplinary care for patients with CKD might be more beneficial in terms of outcomes in the inpatient setting than in the outpatient setting. Furthermore, multidisciplinary care was effective for patients with CKD regardless of whether or not they had diabetes and should be provided at CKD stage G4 at the latest. A multidisciplinary care team should include a nephrologist, a specialist nurse, a physical therapist, and professionals from other fields and is recommended for the management of patients with CKD.

Inpatient education programs have been reported to improve glycemic control, prevent diabetic complications, and reduce hospitalization rates in patients with diabetes (18–20). However, there is little information on the efficacy of multidisciplinary intervention for patients with CKD according to whether the intervention is inpatient-based or outpatient-based. This is the first study to indicate that inpatient multidisciplinary care improves the all-cause mortality risk and initiation of RRT in patients with CKD. Inpatient education programs for patients with CKD have not been implemented extensively in Western countries, probably reflecting differences in the medical insurance system between Japan and Western countries. Although education provided in an outpatient setting is reimbursed for patients with diabetic kidney disease in Japan, it is not reimbursed for patients with other etiologies of CKD. However, full reimbursement is available for these patients if they are admitted to hospital. A few single-center studies in Japan have evaluated the effectiveness of education programs for CKD to date. One study found that the annual rate of decline in eGFR was improved by an inpatient education program, which was continued for 2 years (21). Furthermore, the interval between the start of stage G5 and the start of RRT was longer in patients who received an inpatient education program than in those who did not (22). The patients who received an inpatient education program also had better survival after initiation of dialysis (23). Therefore, multidisciplinary care would be associated with a decreased hospitalization rate, a longer time until initiation of dialysis, and a shorter hospital stay at the start of dialysis, which could lead to a reduction of medical costs. However, the content of the education program and the delivered systems varied according to each facility. Nevertheless, the number of days of hospitalization and the time spent on education should be analyzed. Also, the reasons why it could not be achieved on an outpatient basis should be confirmed. Therefore, further research is required to confirm that the cost-effectiveness of the inpatient setting is superior to that of the outpatient setting.

A meta-analysis revealed that the reduction in all-cause mortality depended on the disciplines represented in the multidisciplinary care team and the stage (24). With only nephrologists and specialist nurses on the team, there was no significant difference in all-cause mortality between patients receiving multidisciplinary care and those who were not. By contrast, when the multidisciplinary care team comprised nephrologists, specialist nurses, and professionals from other disciplines (e.g., dieticians, pharmacists, or social workers), multidisciplinary care was associated with a lower risk of all-cause mortality (25). The FROM-J (Frontier of Renal Outcome Modifications in Japan) study reported that lifestyle and dietary advice provided by a registered dietician in an outpatient setting slowed the rate of deterioration of kidney function in patients with CKD when compared with controls (26). However, the findings were not significant for all stages of CKD and were limited to stage 3; moreover, the multidisciplinary care team comprised only doctors and registered dieticians. In our study, multidisciplinary care was provided by a mean of 4.5 ± 0.6 professionals in the inpatient group and by 2.6 ± 0.7 in the outpatient group. A possible explanation for this result is that when the multidisciplinary care team consists of nephrologists and nurses, the multidisciplinary care model is similar to a conventional model, in which non-multidisciplinary care may be provided by nephrologists and nurses. When the multidisciplinary care group does not include other professionals (e.g., registered dieticians and pharmacists), the education provided for patients with CKD may be insufficient, such that guidelines for dietary protein restriction and other targets are not met, thereby contributing to worsening of kidney function. Patients with CKD require holistic care and support, including dietary modification, maintenance and improvement of medication adherence, education on self-monitoring and early detection of complications, and adequate financial resources to continue treatment. These supports cannot be provided by nephrologists alone and must be implemented by a medical team consisting of multiple professionals. To achieve good outcomes, multidisciplinary care teams that include nephrologists, nurses, registered dieticians, pharmacists, physical therapists, and medical social workers should be involved and have shared goals in terms of individual patients. However, we have no definitive conclusions on how many different cooperating disciplines are needed to achieve optimal outcomes, and further investigations are required to confirm this.

This study has several limitations. First, it did not include a non-multidisciplinary control group. Although multidisciplinary care was not associated with a lower risk of all-cause mortality in previous randomized controlled trials, the risk was found to be reduced in one cohort study (14, 26–28). In addition, the patients could not be randomly allocated to outpatient and inpatient groups because the environment in which multidisciplinary care could be provided varied depending on each facility. Therefore, further prospective randomized controlled trials and large epidemiological studies that include control groups are needed to confirm the efficacy of multidisciplinary care in patients with CKD. Second, we did not investigate changes in blood pressure or laboratory findings other than for kidney function. Salt restriction by multidisciplinary intervention may have lowered blood pressure, reduced proteinuria, and maintained kidney function. We were unable to investigate whether there was any difference in the reduction of salt intake or blood pressure between the study groups. Third, adding or changing medications during the observation period might have affected laboratory findings and kidney function. Renin–angiotensin system inhibitors and sodium-glucose cotransporter-2 inhibitors are recommended for patients with albuminuria, and statins are recommended for all patients with diabetes and CKD (29). Treatment of renal anemia with erythropoiesis-stimulating agents plays an important role in kidney survival (30, 31). Further investigations are needed to determine the contribution of improved adherence with prescribed medication and dietary modification to prevention of worsening kidney function. Finally, there may have been some degree of patient selection and facility bias. Inpatient programs are longer and more expensive than outpatient programs. It is possible that the inpatient group included patients with high self-management ability and a strong desire to prevent progression of their CKD. Therefore, multidisciplinary care in an inpatient setting may be associated with improved patient health literacy. In this study, the participants were divided into two groups by the first intervention method. Therefore, some patients may have been treated in both the inpatient and outpatient settings. Patients might have received multidisciplinary care as an inpatient first, followed by an outpatient setting, or vice versa. However, most facilities in this study provided outpatient or inpatient educational programs based on the hospital functions and human resources. In addition, the content of the education program and the makeup of the patient population varied between the outpatient and inpatient groups from facility to facility. Therefore, the effects of simultaneous participation in outpatient and inpatient sessions should be verified, and educational programs should be standardized to improve the level of care for patients with CKD.

In conclusion, our findings indicate that multidisciplinary care may significantly slow the decline of eGFR, reduce proteinuria in patients with CKD and be effective regardless of diabetes status. Furthermore, this study suggests that multidisciplinary care might be more effective when inpatient-based than when outpatient-based in terms of reducing the all-cause mortality risk and initiation of RRT. Further research is needed to devise a standardized program of multidisciplinary care for both outpatients and inpatients with CKD and to determine which professionals should be involved to achieve the best outcomes for these patients.

## Data availability statement

The raw data supporting the conclusions of this article will be made available by the authors, without undue reservation.

## Ethics statement

The studies involving human participants were reviewed and approved by The ethics committee of Nihon University Itabashi Hospital. Written informed consent for participation was not required for this study in accordance with the national legislation and the institutional requirements.

## Author contributions

MA wrote the manuscript and analyzed the data. TH, YI, TS, and SK designed the study and contributed to data collection. MA, TH, YI, TS, and SK discussed the results and contributed to the final manuscript. All authors read and approved the final manuscript. All authors contributed to the article.
