# Supplementary material for: Inpatient multidisciplinary care can prevent deterioration of renal function in patients with chronic kidney disease: a nationwide cohort study
Source: Front Endocrinol (Lausanne). 2023 Jun 20;14:1180477. doi: 10.3389/fendo.2023.1180477 (PMC10319111; doi:10.3389/fendo.2023.1180477)
Supplement: Supplementary file 1 [file DataSheet_1.docx]

Supplementary Material

Inpatient multidisciplinary care can prevent deterioration of renal function in patients with chronic kidney disease: a nationwide cohort study

Masanori Abe^*^, Tsuguru Hatta, Yoshihiko Imamura, Tsutomu Sakurada, Shinya Kaname

*** Correspondence:** Masanori Abe: [abe.masanori@nihon-u.ac.jp](mailto:abe.masanori@nihon-u.ac.jp)

# Supplementary Table 1. Baseline characteristics of the inpatient group based on the presence or absence of physical therapists

| Variables | Physical therapist (－) | Physical therapist (+) | P-value |
| --- | --- | --- | --- |
| Number of patients, n (% male) | 992 (70.6) | 772 (70.7) | 0.958 |
| Age, years | 70.7 ± 11.7 | 71.8 ± 11.0 | 0.062 |
| Body mass index, kg/m^2^ | 24.4 ± 4.5 | 24.7 ± 4.3 | 0.140 |
| Serum creatinine, mg/dL | 2.10 [1.51–3.07] | 1.88 [1.44–2.79] | 0.001 |
| eGFR, mL/min/1.73m^2^ | 23.1 [15.5–33.9] | 25.9 [17.4–36.3] | 0.001 |
| Serum urea nitrogen, mg/dL | 32 [24–43] | 30 [23–42] | 0.015 |
| Hemoglobin, g/dL | 11.7 ± 1.9 | 11.7 ± 1.9 | 0.829 |
| Serum albumin, g/dL | 3.7 ± 0.6 | 3.7 ± 0.5 | 0.034 |
| Urinary protein, g/gCr | 1.19 [0.28–3.23] | 0.86 [0.17–2.38] | 0.022 |
| CVD comorbidity, n (%) | 279 (28.1) | 233 (30.2) | < 0.0001 |
| Glycated hemoglobin (for diabetes), % | 6.4 ± 1.1 | 6.4 ± 1.1 | 0.901 |
| Primary causes of CKD, n (%) |  |  | 0.003 |
| Diabetic kidney disease | 356 (35.9) | 328 (42.5) |  |
| Nephrosclerosis | 372 (37.5) | 278 (36.0) |  |
| Chronic glomerulonephritis | 160 (16.1) | 89 (11.5) |  |
| Polycystic kidney disease | 30 (3.0) | 12 (1.6) |  |
| Others | 74 (7.5) | 65 (8.4) |  |
| CKD stage, n (%) |  |  | 0.011 |
| G3 (G3a + G3b) | 330 (33.2) | 298 (38.6) |  |
| G3a | 80 (8.0) | 79 (10.2) |  |
| G3b | 250 (25.2) | 219 (28.4) |  |
| G4 | 438 (44.2) | 344 (44.6) |  |
| G5 | 224 (22.6) | 130 (16.8) |  |
| Intervention, days | 8 [6–13] | 7 [6–8] | < 0.0001 |
| Total number of professionals of MDC team, n | 4.2 ± 0.6 | 5.0 ± 0.2 | < 0.0001 |

Data are expressed as number (percentage), mean ± standard deviation, or median [interquartile range] as appropriate. CKD, chronic kidney disease; Cr, creatinine; CVD, cardiovascular disease; eGFR, estimated glomerular filtration rate; MDC, multidisciplinary care.

**2 Supplementary Table 2. Cox proportional hazards models adjusted for confounding factors of all-cause mortality and initiation of renal replacement therapy based on the presence or absence of physical therapists in the inpatient subgroup**

| Group | Unadjusted | | | Model 1 | | | Model 2 | | |
| --- | --- | --- | --- | --- | --- | --- | --- | --- | --- |
|  | HR | 95% CI | P-value | HR | 95% CI | P-value | HR | 95% CI | P-value |
| Physical therapist (－) | 1.00 | Reference | — | 1.00 | Reference | — | 1.00 | Reference | — |
| Physical therapist ( + ) | 0.52 | 0.42–0.63 | <0.0001 | 0.51 | 0.41–0.64 | <0.0001 | 0.55 | 0.42–0.71 | <0.0001 |

Model 1 was adjusted for basic factors, including age, sex, history of cardiovascular diseases, estimated glomerular filtration rate, and urinary protein levels at baseline. Model 2 was adjusted in the same way as Model 1 but with additional adjustments for body mass index, hemoglobin, and serum albumin levels at baseline. CI, confidence interval; eGFR, estimated glomerular filtration rate; HR, hazard ratio.

# 3 Supplementary Table 3. Mean ΔeGFR at each time point in all patients and in the outpatient and inpatient groups

| Variable | All | Outpatient group | Inpatient group | P-value* |
| --- | --- | --- | --- | --- |
| ΔeGFR (-1y) | –5.94 ± 7.52 | –6.09 ± 7.65 | –5.81 ± 7.43 | 0.465 |
| ΔeGFR (+0.5y) | –0.44 ± 5.21 | –0.52 ± 5.23 | –0.40 ± 5.20 | 0.566 |
| ΔeGFR (+1y) | –1.52 ± 6.09 | –1.32 ± 6.01 | –1.63 ± 6.15 | 0.230 |
| ΔeGFR (+2y) | –1.48 ± 3.78 | –1.32 ± 3.64 | –1.56 ± 3.84 | 0.189 |

Data are expressed as the mean ± standard deviation. *P-value for outpatient group vs. inpatient group. ΔeGFR, annual change in eGFR; eGFR, estimated glomerular filtration rate.

## 4 Supplementary Table 4. Mean urinary protein level at each time point in all patients and in the outpatient and inpatient groups

| Variable | All | Outpatient group | Inpatient group | P-value* |
| --- | --- | --- | --- | --- |
| UPCR (Baseline) | 1.09 [0.23–2.98] | 1.20 [0.27–3.25] | 1.01 [0.22–2.87] | 0.218 |
| UPCR (+0.5y) | 1.00 [0.24–2.71] | 1.10 [0.29–2.98] | 0.92 [0.21–2.61] | 0.326 |
| UPCR (+1y) | 0.89 [0.21–2.39] | 0.94 [0.22–2.42] | 0.82 [0.21–2.37] | 0.338 |
| UPCR (+2y) | 0.82 [0.20–2.22] | 0.88 [0.24–2.36] | 0.79 [0.17–2.28] | 0.286 |

Data are expressed as the median [interquartile range]. *P-value for outpatient group vs. inpatient group. UPCR, urinary protein to creatinine ratio.
